# Optimization to Low Temperature Activity in Psychrophilic Enzymes

**DOI:** 10.3390/ijms130911643

**Published:** 2012-09-17

**Authors:** Caroline Struvay, Georges Feller

**Affiliations:** Laboratory of Biochemistry, Centre for Protein Engineering, University of Liège, Institute of Chemistry B6a, B-4000 Liège–Sart Tilman, Belgium; E-Mail: cstruvay@ulg.ac.be

**Keywords:** extremophiles, psychrophiles, cold adaptation, enzyme activity, biotechnology

## Abstract

Psychrophiles, *i.e.*, organisms thriving permanently at near-zero temperatures, synthesize cold-active enzymes to sustain their cell cycle. These enzymes are already used in many biotechnological applications requiring high activity at mild temperatures or fast heat-inactivation rate. Most psychrophilic enzymes optimize a high activity at low temperature at the expense of substrate affinity, therefore reducing the free energy barrier of the transition state. Furthermore, a weak temperature dependence of activity ensures moderate reduction of the catalytic activity in the cold. In these naturally evolved enzymes, the optimization to low temperature activity is reached via destabilization of the structures bearing the active site or by destabilization of the whole molecule. This involves a reduction in the number and strength of all types of weak interactions or the disappearance of stability factors, resulting in improved dynamics of active site residues in the cold. Considering the subtle structural adjustments required for low temperature activity, directed evolution appears to be the most suitable methodology to engineer cold activity in biological catalysts.

## 1. Introduction

Psychrophiles are mainly microorganisms thriving in permanently cold environments and even at sub-zero temperatures in supercooled liquid water. Such extremely cold conditions are encountered, for instance, in salty cryopegs at −10 °C in the permafrost [1] or in the brine veins between polar sea ice crystals at −20 °C [2]. Unusual microbiotopes have also been described, such as porous rocks in Antarctic dry valleys hosting microbial communities surviving at −60 °C [3,4]. These examples illustrate the unsuspected ability of microorganisms to adapt to low temperatures. Such microorganisms do not merely survive or endure such extremely inhospitable conditions but are irreversibly adapted to these environments, as most psychrophiles are unable to grow at mild (or mesophilic) temperatures. It is frequently overlooked that the majority (>80%) of the Earth’s biosphere is cold and permanently exposed to temperatures below 5 °C [5]. Such a low mean temperature mainly arises from the fact that ~70% of the Earth’s surface is covered by oceans that have a constant temperature of 2–4 °C below 1000 m depth, irrespective of the latitude. The polar regions account for another 15%, to which the glacier and alpine regions must be added, as well as the permafrost representing more than 20% of terrestrial soils [6,7]. All these low temperature biotopes have been successfully colonized by cold-adapted organisms, which include a large range of representatives from all three domains: *Bacteria*, *Archaea* and *Eukarya*. As a result, psychrophiles are the most abundant extremophiles in terms of biomass, diversity and distribution.

Life in cold environments requires a vast array of adaptive features at nearly all levels of the cell architecture and function. However, a key determinant of these adaptations lies in the protein function that drives microbial metabolism and cell cycle. Earlier studies of psychrophiles at the molecular level were mainly focused on cold-active enzymes because this aspect was regarded as a prerequisite to the environmental adaptation. It was shown that the high level of specific activity at low temperatures of cold-adapted enzymes is a key adaptation to compensate for the exponential decrease in chemical reaction rates as the temperature is reduced. Such high biocatalytic activity arises from the disappearance of various non-covalent stabilizing interactions, resulting in an improved flexibility of the enzyme conformation [8–10]. As a general picture, psychrophilic enzymes are all faced to a main constraint, to be active at low temperatures, but the ways to reach this goal are quite diverse. It should be noted that this adaptive feature is genetically encoded within the protein sequence and results from a long term selection. We present here an overview of the optimization to low temperature activity in psychrophilic enzymes, also referred to as cold enzymes, and of their biotechnological applications.

## 2. Kinetic Properties of Cold Enzymes

### 2.1. General Properties

The activity of enzymes is strongly dependent on the surrounding temperature. The catalytic constant *k*_cat_ corresponds to the maximum number of substrate molecules converted to product per active site per unit of time, and the temperature dependence of the catalytic rate constant is given by the relation:

(1) $$k_{\text{cat}}=k\frac{k_{\text{B}}T}{h}e^{\frac{\Lambda G^{\#}}{RT}}$$

In this equation, κ is the transmission coefficient generally close to 1, *k*_B_ is the Bolzmann constant (1.38 × 10^−23^ J K^−1^), *h*, the Planck constant (6.63 × 10^−34^ J s), *R*, the universal gas constant (8.31 J K^−1^ mol^−1^) and Δ*G*^#^, the free energy of activation or the variation of the Gibbs energy between the activated enzyme-substrate complex ES* and the ground state ES. Accordingly, the activity *k*_cat_ is exponentially dependent on the temperature. As a rule of thumb, for a biochemical reaction catalyzed by an enzyme from a mesophile (a bacterium or a warm-blooded vertebrate), a drop in temperature from 37 °C to 0 °C results in a 20 to 80 times lower activity. This is the main factor preventing the growth of non-adapted organisms at low temperatures.

**Figure 1:**
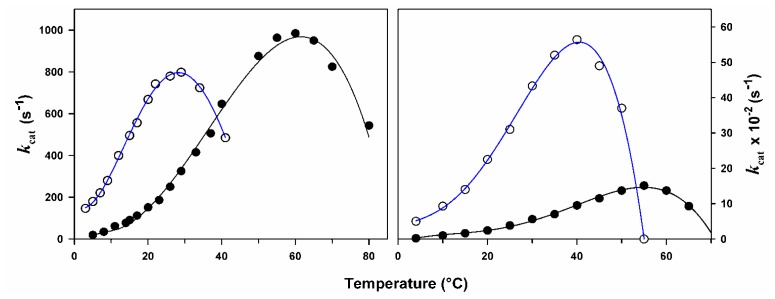
Temperature dependence of activity. The activity of psychrophilic (open symbols, blue lines) and mesophilic (closed symbols) enzymes recorded at various temperatures illustrates the main properties of cold-adapted enzymes: cold activity and heat lability. Left panel, -amylases; right panel, cellulases. Both psychrophilic enzymes are from the Antarctic bacterium *Pseudoalteromonas haloplanktis*. Adapted from [11,12].

The effect of temperature on the activity of psychrophilic and mesophilic enzymes is illustrated in Figure 1. Equation 1 is only valid for the exponential rise of activity with temperature on the left limb of the curves. This figure reveals at least three basic features of cold-adaptation. (*i*) In order to compensate for the slow reaction rates at low temperatures, psychrophiles synthesize enzymes having an up to tenfold higher specific activity in this temperature range. This is in fact the main physiological adaptation to cold at the enzyme level; (*ii*) The temperature for apparent maximal activity for cold-active enzymes is shifted towards low temperatures, reflecting the weak stability of these proteins and their unfolding and inactivation at moderate temperatures; (*iii*) Finally, the adaptation to cold is not always perfect. It can be seen in Figure 1 (left panel) that the specific activity of the psychrophilic enzymes at low temperatures, although very high, remains lower than that of the mesophilic enzyme at 37 °C.

### 2.2. Heat-Labile and Unstable Cold Enzymes

**Figure 2:**
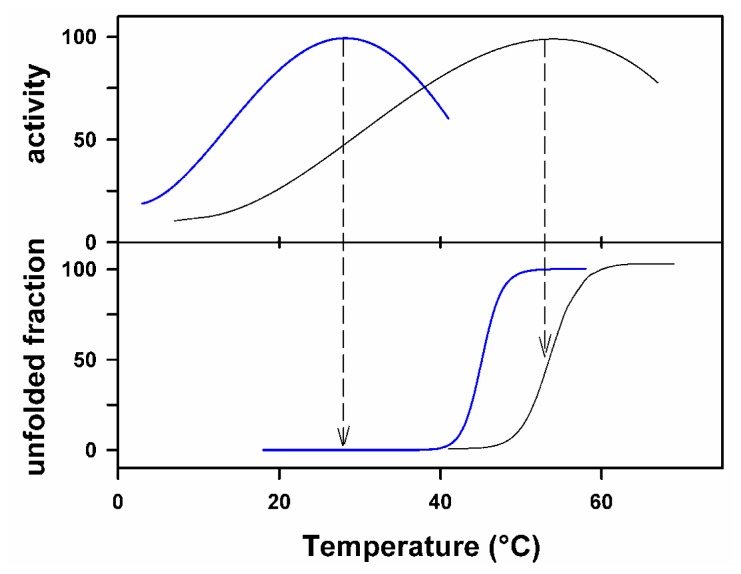
Inactivation and unfolding of psychrophilic enzymes. The activity of psychrophilic enzymes (upper panel, blue line) is inactivated by temperature before unfolding of the protein structure (lower panel, blue line) illustrating the pronounced heat-lability of the active site. By contrast, inactivation of mesophilic enzymes (black curves) closely corresponds to the loss of the protein conformation. Adapted from [14].

Most psychrophilic enzymes share at least one property: a heat-labile activity, irrespective of the protein structural stability. Furthermore, the active site appears to be the most heat-labile structural element of these proteins [13–15]. Figure 2 illustrates this significant difference between the stability of the active site and the stability of the structure. The lower panel shows the stability of the structure as recorded by fluorescence. As expected, the structure of the cold-active enzyme is less stable than the mesophilic one. In the upper panel, the activity is recorded under the same experimental conditions and it can be seen that the mesophilic enzyme is inactivated when the protein unfolds. By contrast, activity of the cold-active enzyme is lost before the protein unfolds. This means that the active site is even more heat-labile than the whole protein structure. It was also shown that the active site of a psychrophilic -amylase is the first structural element that unfolds in transverse urea gradient gel electrophoresis [16]. All these aspects point to a very unstable and flexible active site and illustrate a central concept in cold adaptation: localized increases in flexibility at the active site are responsible for the high but heat-labile activity [17], whereas other regions of the enzyme might or might not be characterized by low stability when not involved in catalysis. For instance, psychrophilic carbonic anhydrase [18] and isocitrate dehydrogenase [19] are highly stable enzymes with however improved flexibility in regions driving catalysis.

Beside this general rule, some rare exceptions have been reported so far. The chaperonin and heat-shock protein GroEL from an Antarctic bacterium is not cold-adapted and displays similar stability and activity than that of its *E. coli* homologue [20]. It has been suggested that this chaperonin remains suited to function during sudden temperature increases of the environment [21]. Similarly, activity of thioredoxin from the same bacterium is much more heat-stable than that of *E. coli* [22]. One cannot exclude the possibility that enzymes involved in electron transfer do not require the same type of adaptations because the rate of electron flow is not significantly affected by the low biological temperatures.

### 2.3. Active Site Structure

**Figure 3:**
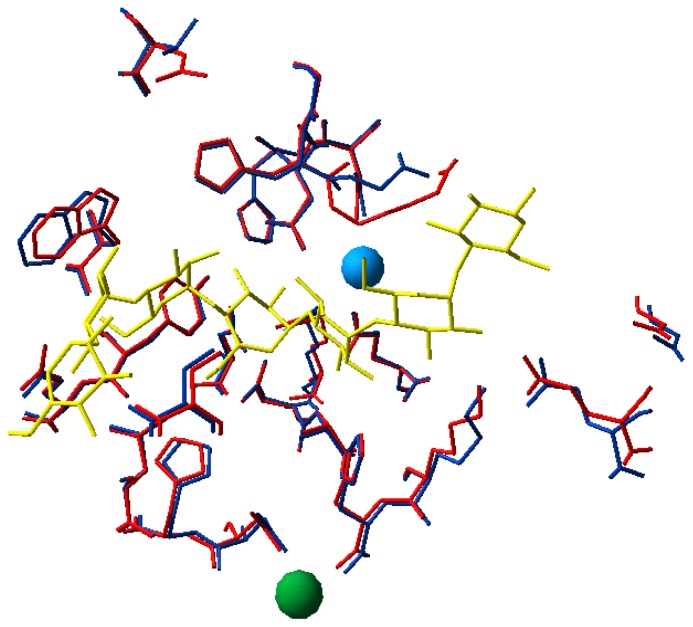
Structure of the active site. Superimposition of the active site residues in psychrophilic (blue) and mesophilic α-amylases (red). The chloride and calcium ions are shown as blue and green spheres, respectively. The 24 residues performing direct or water-mediated interactions with the substrate analog derived from acarbose (yellow) are identical and superimpose perfectly within the resolution of the structures, demonstrating a structural identity in these psychrophilic and mesophilic enzymes [27].

Crystal structures of psychrophilic enzymes were of course of prime importance to investigate the properties of these heat-labile and cold-active catalytic centers. The first basic observation is that all side chains involved in the catalytic mechanism are strictly conserved. Indeed, comparison of the first X-ray structure of a psychrophilic enzyme, the cold-active -amylase [23,24], and of its closest structural homologue from pig both in complex with acarbose, a pseudosaccharide inhibitor mimicking the transition state intermediate [25,26], has shown that all 24 residues forming the catalytic cleft are strictly conserved in the cold-active α-amylase (Figure 3). This outstanding example of active site identity demonstrates that the specific properties of psychrophilic enzymes can be reached without any amino acid substitution in the reaction center. As a consequence, changes occurring elsewhere in the molecule are responsible for the optimization of the catalytic parameters (see Section 4).

Nevertheless, significant structural adjustments at the active site of psychrophilic enzymes have been frequently reported. In many cases, a larger opening of the catalytic cleft is observed and achieved by various ways, including replacement of bulky side chains for smaller groups, distinct conformation of the loops bordering the active site or small deletions in these loops, as illustrated by a cold active citrate synthase [28]. In the case of a Ca^2+^, Zn^2+^-protease from a psychrophilic *Pseudomonas* species, an additional bound Ca^2+^ ion pull the backbone forming the entrance of the site and markedly increases its accessibility when compared with the mesophilic homologue [29]. As a result of such a better accessibility, cold-active enzymes can accommodate substrates at lower energy cost, as far as the conformational changes are concerned, and therefore reduce the activation energy required for the formation of the enzyme-substrate complex. The larger active site may also facilitate easier release and exit of products and thus may alleviate the effect of a rate limiting step on the reaction rate.

In addition, differences in electrostatic potentials in and around the active site of psychrophilic enzymes appear to be a crucial parameter for activity at low temperatures. Electrostatic surface potentials generated by charged and polar groups are an essential component of the catalytic mechanism at various stages: as the potential extends out into the medium, a substrate can be oriented and attracted before any contact between enzyme and substrate occurs. Interestingly, the cold-active citrate synthase [28], malate dehydrogenase [30], uracyl-DNA glycosylase [31] and trypsin [32–34] are characterized by marked differences in electrostatic potentials near the active site region compared to their mesophilic or thermophilic counterparts that may facilitate interaction with ligand. In the case of malate dehydrogenase for example, the increased positive potential at and around the oxaloacetate binding site and the significantly decreased negative surface potential at the NADH binding region may facilitate the interaction of the oppositely charged ligands with the surface of the enzyme [30]. In all cases, the differences were caused by discrete substitutions in non-conserved charged residues resulting in local electrostatic potential differing in both sign and magnitude.

### 2.4. Active Site Dynamics

The heat-labile activity of psychrophilic enzymes suggests that the dynamics of the functional side chains at the active site is improved in order to contribute to cold-activity and the above mentioned structural adaptations seem to favor a better accessibility to the substrate and release of the product. This view is strongly supported by the enzymological properties of cold-active enzymes. Non-specific psychrophilic enzymes accept various substrates and have a broader specificity than the mesophilic homologues, because substrates with slightly distinct conformations or sizes can fit and bind to the site. For instance, the observed differences in substrate specificity between Atlantic salmon and mammalian elastases have been interpreted to be based on a somewhat wider and deeper binding pocket for the cold-adapted elastase [32]. The broad specificity of a psychrophilic alcohol dehydrogenase that oxidizes large bulky alcohols was also assigned to a highly flexible active site [35]. Several crystal structures of psychrophilic enzymes also point to an increased flexibility at or near the active site [36], as also supported by molecular dynamic simulations [37–40].

This active site flexibility of cold-active enzymes in solution is also well demonstrated by the psychrophilic α-amylase [41]. As shown in Table 1, both the psychrophilic and mesophilic α-amylases degrade large macromolecular polysaccharides made of glucose units linked by -1,4 bonds. These substrates have a complex structure and are generally branched. Taking the natural substrate, starch, as the reference, it can be seen that the psychrophilic enzyme is more active on all these large substrates. Being more flexible, the active site can accommodate easily these macromolecular polysaccharides. Considering the small substrates, the reverse situation is observed. Both enzymes are active on short oligosaccharides of at least four glucose units but in this case, the psychrophilic α-amylase is less active on all these small substrates. Apparently, the flexible active site accommodates less efficiently these short oligosaccharides.

### 2.5. Adaptive Drift of Substrate Affinity

As a consequence of the improved active site dynamics in cold-active enzymes, substrates bind less firmly in the binding site (if no point mutations have occurred) giving rise to higher *K*_m_ values. An example is given in Table 2 showing that the psychrophilic α-amylase is more active on its macromolecular substrates whereas the *K*_m_ values are up to 30-fold larger, *i.e*., the affinity for the substrates is up to 30-fold lower. Ideally, a functional adaptation to cold would mean optimizing both *k*_cat_ and *K*_m_. However, a survey of the available data on psychrophilic enzymes indicates that optimization of the *k*_cat_/*K*_m_ ratio is far from a general rule but on the contrary that the majority of cold-active enzymes improve the *k*_cat_ value at the expense of *K*_m_, therefore leading to suboptimal values of the *k*_cat_/*K*_m_ ratio, as also shown in Table 2. For instance, high *K*_m_ values have been reported for psychrophilic aspartate carbamoyltransferase [42,43], triose-phosphate isomerase [44], subtilisin [45], lactate dehydrogénase [17,46], DNA ligase [47], elongation factor Tu [48] and G [49], glutamate dehydrogenase [50,51], α-amylase [52], dihydrofolate reductase [53], cellulase [54], endonuclease I [55], aspartate aminotransferase [56], isocitrate dehydrogenase [57], xylanase [58], ornithine carbamoyltransferase [59], citrate synthase [60], purine nucleoside phosphorylase [61], DEAD-Box proteins [62] and acetate kinase [63].

There is in fact an evolutionary pressure on *K*_m_ to increase in order to maximize the overall reaction rate. Such adaptive drift of *K*_m_ has been well illustrated by the lactate dehydrogenases from Antarctic fish [17] and by the psychrophilic α-amylase [52] because both enzymes display rigorously identical substrate binding site and active site architecture when compared with their mesophilic homologues. In both cases, temperature-adaptive increases in *k*_cat_ occur concomitantly with increases in *K*_m_ in cold-active enzymes. As already mentioned, such identity of the sites also implies that adjustments of the kinetic parameters are obtained by structural changes occurring distantly from the reaction center. This aspect has received strong experimental supports [64,65].

### 2.6. Adaptive Optimization of Substrate Affinity

Several enzymes, especially in some cold-adapted fish, counteract this adaptive drift of *K*_m_ in order to maintain or to improve the substrate binding affinity by amino acid substitutions within the active site [32,66]. The first reason for these enzymes to react against the drift is obvious when considering the regulatory function associated with *K*_m_, especially for intracellular enzymes. The second reason is related to the temperature dependence of weak interactions. Substrate binding is an especially temperature-sensitive step because both the binding geometry and interactions between binding site and ligand are governed by weak interactions having sometimes opposite temperature dependencies. Hydrophobic interactions form endothermically and are weakened by a decrease in temperature. By contrast, interactions of electrostatic nature (ion pairs, hydrogen bounds, Van der Waals interactions) form exothermically and are stabilized at low temperatures. Therefore low temperatures do not only reduce the enzyme activity (*k*_cat_), but can also severely alter the substrate binding mode according to the type of interaction involved.

## 3. Thermodynamic Origin of Optimization

**Figure 4:**
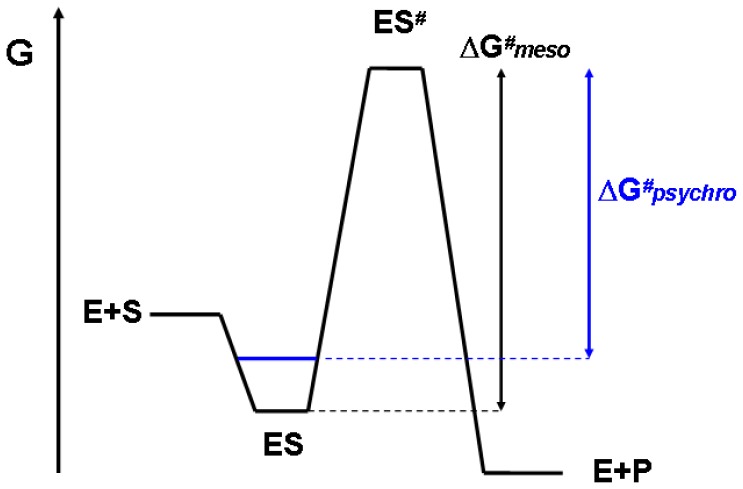
Optimization of activity by decreasing substrate affinity in psychrophilic enzymes. Reaction profile for an enzyme-catalyzed reaction with Gibbs energy changes under saturating substrate concentration. Weak substrate binding (in blue) decreases the activation energy (Δ*G^#^psychro*) and thereby increases the reaction rate. In this scheme, the energy levels of E + S and of ES*^#^* are assumed to be similar [10].

Referring to Equation 1, the high activity of cold-adapted enzymes corresponds to a decrease of the free energy of activation Δ*G^#^*. Two strategies have been highlighted to reduce the height of this energy barrier. Figure 4 illustrates the first strategy where an evolutionary pressure increases *K*_m_ in order to maximize the reaction rate. According to the transition state theory, when the enzyme encounters its substrate, the enzyme-substrate complex ES falls into an energy pit. For the reaction to proceed, an activated state ES*^#^* has to be reached, that eventually breaks down into the enzyme and the product. The height of the energy barrier between the ground state ES and the transition state ES*^#^* is defined as the free energy of activation Δ*G^#^*: the lower this barrier, the higher the activity as reflected in Equation 1. In the case of cold active enzymes displaying a weak affinity for the substrate, the energy pit for the ES complex is less deep (dashed in Figure 4). It follows that the magnitude of the energy barrier is reduced and therefore the activity is increased. This thermodynamic link between affinity and activity is valid for most enzymes (extremophilic or not) under saturating substrate concentrations and this link appears to be involved in the improvement of activity at low temperatures in numerous cold-active enzymes [17,59].

The second and more general strategy involves the temperature-dependence of the reaction catalyzed by cold-active enzymes. Table 3 reports the enthalpic and entropic contributions to the free energy of activation in extremophilic α-amylases. The free energy of activation Δ*G^#^* is calculated from Equation 1 using the *k*_cat_ value at a given temperature and the enthalpy of activation Δ*H^#^* is obtained by recording the temperature dependence of the activity [67]. Finally, the entropic contribution *T*Δ*S^#^* is deduced from the Gibbs-Helmholtz equation:

(2) $$\Delta G^{\#}=\Delta H^{\#}-T\Delta S^{\#}$$

The enthalpy of activation Δ*H^#^* depicts the temperature dependence of the activity: the lower this value, the lower the variation of activity with temperature. The low value found for almost all psychrophilic enzymes demonstrates that their reaction rate is less reduced than for other enzymes when the temperature is lowered. Accordingly, the decrease of the activation enthalpy in the enzymatic reaction of psychrophilic enzymes can be considered as the main adaptive character to low temperatures. This decrease is structurally achieved by a decrease in the number of enthalpy-driven interactions that have to be broken during the activation steps. These interactions also contribute to the stability of the protein folded conformation and, as a corollary, the structural domain of the enzyme bearing the active site should be more flexible. It is interesting to note that such a macroscopic interpretation of the low activation enthalpy in cold-active enzymes fits with the experimental observation of a markedly heat-labile activity illustrated in Figure 2. Table 3 shows that the entropic contribution *T*Δ*S^#^* for the cold-active enzyme is larger and negative. This has been interpreted as a large reduction of the apparent disorder between the ground state with its relatively loose conformation and the well organized and compact transition state [67]. The heat-labile activity of cold-active enzymes suggests a macroscopic interpretation for this thermodynamic parameter. As a consequence of active site flexibility, the enzyme-substrate complex ES occupies a broader distribution of conformational states translated into increased entropy of this state, compared to that of the mesophilic or thermophilic homologues. This assumption has received strong experimental support by using microcalorimetry to compare the stabilities of free extremophilic enzymes with the same enzymes trapped in the transition state conformation by a non-hydrolysable substrate analog [14]. The larger increase in stability for the psychrophilic enzyme in the transition state conformation demonstrated larger conformational changes between the free and bound states when compared to mesophilic and thermophilic homologues. Furthermore, a broader distribution of the ground state ES should be accompanied by a weaker substrate binding strength, as indeed observed for numerous psychrophilic enzymes. Finally, it should be mentioned that the typical activation parameters of psychrophilic enzymes are well reproduced by reaction kinetic simulations [68].

## 4. Structural Origin of Cold Activity

Many observations similar to Figure 1 have suggested relationships between the activity of the enzyme, the flexibility of the protein and its stability. Indeed, the high activity at low temperatures seems to arise from an increased flexibility of the protein structure, especially at temperatures that strongly slow down molecular motions, but the consequence of this improved mobility of the protein structure is of course a weak stability. As a matter of fact, various biophysical studies using fluorescence quenching [13–15] or neutron scattering [69] have revealed a less compact conformation of psychrophilic enzymes, undergoing frequent micro-unfolding events.

The number of X-ray crystal structures from psychrophilic enzymes has increased dramatically, demonstrating the growing interest for these peculiar proteins. However, the interpretation of these structural data is frequently difficult for two main reasons. First, the structural adaptations are extremely discrete and can easily escape the analysis. Second, these structural adaptations are very diverse, reflecting the complexity of factors involved in the stability of a macromolecule at the atomic level. For instance, it was found that all structural factors currently known to stabilize the protein molecule could be attenuated in strength and number in the structure of cold-active enzymes [32,70–72]. An exhaustive description of all these factors is beyond the scope of this chapter and only the essential features are summarized below. Two review articles can be consulted for a comprehensive discussion of this topic [9,32].

The observable parameters related to protein stability include structural factors and mainly weak interactions between atoms of the protein structure. In psychrophilic proteins, this involves the clustering of glycine residues (providing local mobility) [73,74], the disappearance of proline residues in loops (enhancing chain flexibility between secondary structures) [75], a reduction in arginine residues which are capable of forming multiple salt bridges and H-bonds, as well as a lower number of ion pairs, aromatic interactions or H-bonds, compared to mesophilic enzymes. The size and relative hydrophobicity of non-polar residue clusters forming the protein core are frequently smaller, lowering the compactness of the protein interior by weakening the hydrophobic effect on folding. Psychrophilic proteins have larger cavity sizes sufficient to accommodate water molecules: these cavities and embedded water molecules can play a significant role in structural flexibility [76]. The *N* and *C*-caps of α-helices are also altered (weakening the charge-dipole interaction) and loose or relaxed protein extremities appear to be preferential sites for unzipping. The binding of stabilizing ions, such as calcium, can be extremely weak, with binding constants differing from mesophiles by several orders of magnitude. Insertions and deletions are sometimes responsible for specific properties such as the acquisition of extra-surface charges (insertion) or the weakening of subunit interactions (deletion).

Calculation of the solvent accessible area showed that some psychrophilic enzymes expose a higher proportion of non-polar residues to the surrounding medium [24,28]. This is an entropy-driven destabilizing factor caused by the reorganization of water molecules around exposed hydrophobic side chains. Calculations of the electrostatic potential revealed in some instances an excess of negative charges at the surface of the protein and, indeed, the pI of cold-active enzymes is frequently more acidic than that of their mesophilic or thermophilic homologues. This has been related to improved interactions with the solvent, which could be of prime importance in the acquisition of flexibility near zero degrees [77]. Besides the balance of charges, the number of salt bridges covering the protein surface is also reduced. There is a clear correlation between surface ion pairs and temperature adaptation, since these weak interactions significantly increase in number from psychrophiles to mesophiles, to thermophiles and hyperthermophiles, the latter showing arginine-mediated multiple ion pairs and interconnected salt bridge networks [78,79]. Such an altered pattern of electrostatic interactions is thought to improve the dynamics or the “breathing” of the external shell of cold-active enzymes.

However, each enzyme adopts its own strategy by using one or a combination of these altered structural factors in order to improve the local or global mobility of the protein edifice. Accordingly, a general theory for structural adaptations cannot be formulated but nevertheless, enzyme families sharing the 3D fold can be compared [80]. Comparative structural analyses of psychrophilic, mesophilic and thermophilic enzymes indicate that each protein family displays different structural strategy to adapt to temperature [71,80–85]. However, some common trends are observed: the number of ion pairs, the side-chain contribution to the exposed surface, and the apolar fraction of the buried surface show a consistent decrease with decreasing optimal temperatures [71]. The multitude of structural strategies in cold-adapted proteins has also complicated statistical analyses aimed at delineating general trends in temperature adaptation. Various trends have been reported for psychrophilic proteins using different methodologies and datasets: a preference for smaller-size and less hydrophobic residues [86]; a less hydrophobic core, less charged and long-chained surface residues [87]; a decrease of solvent accessible surface contribution of charged residues and an increase of hydrophobic surface contribution [88] or various patterns of preferential amino acid substitutions [89,90].

## 5. Biotechnological Usefulness of Psychrophilic Enzymes

As mentioned in previous sections, most enzymes from psychrophiles are cold-active and heat-labile. Psychrophilic enzymes can be up to ten times more active at low and moderate temperatures as compared with their mesophilic homologues. Furthermore, psychrophilic enzymes are frequently inactivated at temperatures that are not detrimental for their mesophilic counterparts. These specific traits are responsible for the three main advantages of cold-active enzymes in biotechnology: (*i*) as a result of their high activity, a lower concentration of the enzyme catalyst is required to reach a given activity, therefore reducing the amount of costly enzyme preparation in a process; (*ii*) as a result of their cold-activity, they remain efficient at tap water or ambient temperature, therefore avoiding heating during a process, either at domestic (e.g., washing machine) or industrial levels; and (*iii*) as a result of heat-lability, they can be efficiently and sometime selectively inactivated after a process by moderate heat input. Beside these traits specifically linked to temperature adaptation, an additional important aspect has to be mentioned: enzymes from organisms endemic to cold environments can be a valuable source of new catalysts possessing useful enzymological characteristics such as novel substrate specificities or product properties, as exemplified by lipases from the yeast *Candida antarctica* or by the xylanase from the bacterium *Pseudoalteromonas haloplanktis* (see below). Previous reviews should be consulted for a complete coverage of this topic [7,91–97]. Bioprospector, an online database [98] provides a survey of patents, commercial products and companies involved in applied research using genetic resources from both the Antarctic and the Arctic. This excellent initiative, accompanied by relevant publications, is currently the most updated survey of biotechnological applications based on psychrophiles and on their biomolecules. Some specific examples are provided below.

### 5.1. Molecular Biology

In a pioneering work, Kobori *et al.* [99] have purified and characterized a heat-labile alkaline phosphatase from an Antarctic bacterium isolated in McMurdo Sound. Alkaline phosphatases are mainly used in molecular biology for the dephosphorylation of DNA vectors prior to cloning to prevent recircularization, for the dephosphorylation of 5′-nucleic acid termini before 5′-end labeling by polynucleotide kinase or for removal of dNTPs and pyrophosphate from PCR reactions. However, the phosphatase has to be carefully removed after dephosphorylation to avoid interferences with the subsequent steps. Furthermore, *E. coli* and calf intestinal alkaline phosphatase (that was the preferred enzyme for these applications) are heat-stable and require detergent addition for inactivation. It follows that heat-labile alkaline phosphatases are excellent alternatives as they are inactivated by moderate heat treatment allowing to perform the subsequent steps in the same test tube and minimizing nucleic acid losses. While the scientific report of Kobori *et al.* [99] specifically stressed the usefulness of their heat-labile alkaline phosphatase as a new tool in molecular biology, this interesting finding was apparently not turned into a marketed product. Fifteen years later, the group of V. Bouriotis isolated an alkaline phosphatase from another Antarctic bacterium and cloned its gene in *E. coli* [100], solved its crystal structure [101] and also showed that its properties can be further improved by directed evolution in terms of high activity and heat-lability [102]. This heat-labile alkaline phosphatase, sold as Antarctic phosphatase, is now proposed on the market by New England Biolabs Inc. (Ipswich, MA, USA). In the same context, the heat-labile alkaline phosphatase from the Arctic shrimp *Pandalus borealis* is also available for instance from Biotec Pharmacon ASA (Tromsø, Norway) or GE Healthcare Life Sciences (Little Chalfont, UK).

Two other psychrophilic enzymes are also marketed for molecular biology applications taking advantage of the heat-labile property. Shrimp nuclease selectively degrades double stranded DNA: for instance, it is used for the removal of carry-over contaminants in PCR mixtures, and then it is heat-inactivated prior addition of the template. This enzyme is produced in recombinant form in *Pichia pastoris* and is available from Biotec Pharmacon ASA (Tromsø, Norway), USB Corporation (Santa Clara, CA, USA) or Thermo Scientific (Waltham, MA, USA). Heat-labile uracil-DNA *N*-glycosylase from Atlantic cod (*Gadus morhua*), that presents typical cold adaptation features [31], is also used to remove DNA contaminants in sequential PCR reactions. When PCR is performed with dUTP instead of dTTP, PCR products become distinguishable from target DNA, and can be selectively degraded by uracil-DNA *N*-glycosylase. Following degradation of contaminants, the enzyme is completely and irreversibly inactivated after heat treatment. Heat-labile uracil-DNA *N*-glycosylase, produced in recombinant form in *E. coli*, is available from Biotec Pharmacon ASA (Tromsø, Norway).

### 5.2. Industrial Enzymes

At the industrial level, the best-known representative of polar microorganisms is certainly the yeast *Candida antarctica*, as its species name unambiguously refers to the sampling origin. This yeast produces two lipases, A and B, the latter being sold for instance as Novozym 435 by Novozymes (Bagsvaerd, Denmark). Although the moderate heat-stability of this lipase in aqueous solutions can be of concern, this enzyme is stabilized in its immobilized form. As a result of its substrate and stereospecificity, lipase B is involved in a very large number of organosynthesis applications related to food/feed processing, pharmaceuticals or cosmetics [103]. In a survey of patents related to Antarctica [104] it was shown that lipases from *C. antarctica* by far dominate the number of process- or product-based patents. This is a significant example of the potential for novel catalysts from genetic resources in cold environments.

The market for enzymes used in detergents represents 30%–40% of all enzymes produced worldwide. Amongst these enzymatic cleaning agents, subtilisin (an alkaline serine protease predominantly produced by *Bacillus* species) largely dominates this market. At the domestic level, the current trend is however to use detergents at lower washing temperatures because of the associated reductions in energy consumption and costs as well as to protect texture and colors of the fabrics. Accordingly, cold-active subtilisins are required for optimal washing results at tap water temperatures and the current advertisements for cold-active detergents indicate that this goal has been reached. The first psychrophilic subtilisins isolated from Antarctic *Bacillus* species have been extensively characterized to comply with such requirement [45,105]. However, they suffered from a low heat-stability that can compromise their storage but also from a low chemical stability towards the detergent components. Therefore, subtilisins currently incorporated in cold-active detergents are engineered enzymes that combine storage stability, alkaline stability and activity and cold-activity. Although psychrophilic subtilisins are not components *per se* of cold-active detergents, they have largely contributed to the advancement of this economically attractive concept.

### 5.3. Food Technology

The xylanase from the Antarctic bacterium *Pseudoalteromonas haloplanktis* is a nice example of the successful biotechnological transfer to the food industry. Xylanases are glycoside hydrolases that degrade the polysaccharide beta-1,4-xylan, thus breaking down hemicellulose, one of the major components of plant cell walls. Xylanases are also a key ingredient of industrial dough conditioners used to improve bread quality. It was found that the Antarctic enzyme belonged to a new class of xylanases as both its amino acid sequence and fold were distinct from previously characterized xylanases. The psychrophilic enzyme was therefore subjected to intensive investigations aimed at elucidating the structural origins of its high cold activity and weak stability as well as at understanding its enzymological mode of action [13,58,106–108]. Furthermore, baking trials have revealed that the psychrophilic xylanase was very effective in improving the dough properties and final bread quality with, for instance, a positive effect on loaf volume [109]. This efficiency appears to be related to the high activity of the psychrophilic xylanase at cool temperatures required for dough resting and to its specific mode of xylan hydrolysis. Following careful production optimization of this peculiar xylanase, the product is now sold by Puratos (Grand-Bigard, Belgium). This is apparently the psychrophilic enzyme produced at the highest amounts to date.

Beta-galactosidase, or lactase, is also a glycoside hydrolase that specifically hydrolyzes the milk sugar lactose into galactose and glucose. It should be stressed that 75% of the world population suffers from lactose intolerance arising from deficient synthesis of intestinal lactase in adults and resulting in digestive disorders due to fermentation of lactose by enteric bacteria. In this context, a cold-active lactase from an Antarctic bacterium has been patented (WO 01/04276A1) for its capacity to hydrolyze lactose during milk storage at low temperatures [110]. It is worth mentioning that commercially available lactases require milk heating to become active. This heating step has however detrimental effects on milk quality as it alters the aspect, the taste and texture (Maillard reactions, activation of proteases, coagulation, …). Although the psychrophilic lactase is apparently not used for this specific application, it is expected that it will be produced soon in large quantities by Nutrilab NV (Bekkevoort, Belgium) to hydrolyze lactose (a by-product of the dairy industry) in the process of the high value sweetener d-tagatose, a natural monosaccharide with low caloric value and glycemic index.

### 5.4. Engineering Cold Activity

When considering the biotechnological potential of cold-active and/or heat-labile enzymes, it is tempting to devise a mutational strategy in order to introduce these properties into an already well characterized commercial enzyme. However, engineering psychrophilic activity in a mesophilic enzyme by rational design has not been reported to date. The main reason should be found in the huge complexity of amino acid substitutions and interactions leading to psychrophilic activity that have been optimized during evolution on a long timescale. Accordingly, engineering psychrophilic activity currently escape our computational capacity. By contrast, laboratory evolution, that mimics natural selection, has been successful in producing cold-active enzymes with or without heat-lability, as reviewed in [111]. It is worth mentioning that, in many cases, the effect of the selected random mutations cannot be properly explained, underlining again the complexity of the structural factors responsible for psychrophilic activity. The possibility to introduce several random mutations in a gene, to combine these mutations and to screen a large number of mutants for a specific property by directed evolution seems to be the best strategy to engineer enzyme cold-activity. Improved cold-activity by chemical modification has also been reported [112–114] but, although sometimes successful, the results of chemical modification remain unpredictable.

## 6. Conclusions

Cold-active enzymes are a key determinant in psychrophiles’ adaptation to life at low temperature. We have depicted the latest research on psychrophilic enzymes, underlining that cold-activity is not a uniform property: the extent of low temperature activity is variable, the adaptive optimization of the kinetic parameters differs in amplitude and origin and the structural factors involved are diverse and complex. Obviously, fine enzymological studies are required to improve our understanding of these essential adaptations for the vast range of organisms thriving on our cold planet. The biotechnological applications of these cold-active and heat-labile enzymes are still in their infancy. Nevertheless, future developments of these biocatalysts and of their derivatives are expected because they are frequently involved in environmentally friendly processes and contribute to energy saving, both aspects being of increasing significance.

## Figures and Tables

**Table 1:** Relative activity of the psychrophilic (AHA) and the mesophilic (PPA)-amylases on macromolecular polysaccharides and on maltooligasaccharides. Adapted from [41].

Substrate	Relative activity (%)	

	AHA	PPA
Macromolecular substrates		
Starch	100	100
Amylopectin	96	68
Amylose	324	214
Dextrin	108	95
Glycogen	74	59
Short oligosaccharides		
Maltotetraose G4	17	22
Maltopentose G5	69	145
Maltohexaose G6	94	147
Maltoheptaose G7	119	155
Maltooligosaccharides (G4 to G10 mix)	64	101

**Table 2:** Kinetic parameters for the hydrolysis of polysaccharides at 25 °C by the psychrophilic (AHA) and the mesophilic (PPA) α-amylases. Adapted from [41].

	AHA			PPA		
		
Substrate	*k*_cat_ s^−1^	*K*_m_ mg L^−1^	*k*_cat_*/K*_m_ s^−1^mg^−1^ L	*k*_cat_ s^−1^	*K*_m_ mg L^−1^	*k*_cat_*/K*_m_ s^−1^mg^−1^ L
Starch	663	155	4.3	327	41	8.0
Amylopectin	636	258	2.5	222	53	4.2
Amylose	2148	178	12.1	700	36	19.4
Dextrin	716	586	1.2	311	61	5.1
Glycogen	491	1344	0.3	193	46	4.2

**Table 3:** Activation parameters of the hydrolytic reaction of α-amylases at 10 °C. Adapted from [14].

	*k_cat_* s^−1^	Δ*G^#^* kcal mol^−1^	Δ*H^#^* kcal mol^−1^	*T*Δ*S^#^* kcal mol^−1^
Psychrophile	294	13.3	8.3	−5.0
Mesophile	97	14.0	11.1	−2.9
Thermophile	14	15.0	16.8	1.8
